# A Prospective Comparative Study of Laparoscopic Totally Extraperitoneal (TEP) and Laparoscopic Transabdominal Preperitoneal (TAPP) Inguinal Hernial Repair

**DOI:** 10.7759/cureus.42209

**Published:** 2023-07-20

**Authors:** Ratnesh K Jaiswal, N K Pandey, Aditya Tolat, Dheer S Kalwaniya, Amit K Gupta, Vakulabharanam Naga Rohith, Pawan Gurivelli, Reena Meena

**Affiliations:** 1 General Surgery, Vardhman Mahavir Medical College and Safdarjung Hospital, New Delhi, IND; 2 General Surgery, Asian Institute of Medical Sciences, Faridabad, IND

**Keywords:** tep, laparoscopy, mesh repair, tapp, hernia, inguinal

## Abstract

Introduction

Inguinal hernia is a common surgical problem throughout the world. Currently, the management options available are open mesh hernioplasty and laparoscopic mesh repair. Laparoscopic mesh repair can be performed by either transabdominal preperitoneal (TAPP) repair or totally extraperitoneal (TEP) repair. Many studies comparing the two procedures have been unable to establish the superiority of one procedure over the other and have yielded conflicting results. Thus, we performed this study to compare TAPP and TEP.

Aim

The aim of this study is to compare the clinical outcomes and safety of laparoscopic TEP and laparoscopic TAPP for inguinal hernia repair.

Materials and methods

Patients were randomly divided into two groups on the basis of surgical procedures. The first group of patients underwent laparoscopic TAPP mesh repair, and the second group of patients underwent laparoscopic TEP mesh repair. Their intraoperative and postoperative findings were noted. Patients were followed up at regular intervals for up to six months.

Results

The mean age and mean weight distribution between the two groups were not significant. The duration of surgery needed (in minutes) for TAPP was found to be significantly less compared to TEP. In the TEP group, conversion to open occurred for three subjects (6.7%) while there was no conversion in the TAPP group. Postoperative pain at 24 hrs was found to be higher in TAPP subjects compared to that in TEP subjects, but the difference was statistically insignificant. Tolerance to a liquid diet started few hours after surgery was found to be the same in both groups. Association of the duration of hospital stays with the type of surgery was not significant. Six subjects (13.2%) showed hematoma in the TEP group while five subjects (11%) in the TAPP group showed hematoma after one week of surgery. Eight subjects (17.6%) showed seroma in the TEP group while three subjects (15.4%) in the TAPP group showed seroma after one week of surgery. Two subjects (4.4%) showed superficial wound infection in both the TEP group and TAPP group after one week of surgery. Four subjects each (8.9%) showed scrotal edema in the TEP group as well as the TAPP group after one week of surgery. No subject showed port site hernia without closure of the sheath at one-week, one-month, and six-month follow-up visits. Two subjects (4.4%) each showed groin pain in the TEP group as well as the TAPP group after one week of surgery. There were no instances of bowel obstruction or mesh infection.

Conclusion

TEP is a more skill-demanding procedure as compared to TAPP and thus takes more time to perform. However, it is superior on account of not breaching the peritoneum. TAPP is favorable for larger hernias. The choice of procedure should be individualized according to the patient's characteristics and surgeon's preference.

## Introduction

An inguinal hernia is a protrusion of abdominal cavity contents through the inguinal canal. This is the most common type of hernia, and it mainly affects men. It is said to be often associated with aging and repeated strain on the abdomen [1]. Hernia is a common problem in the modern world with an incidence ranging from 5% to 7%. The prevalence of hernia is far greater in developing countries like India as the occupations of many people are farming, hauling construction, lifting weights, etc. amounting to a major health care burden. Of all groin hernias, around 75% are inguinal hernias [2,3].

In developing countries, a considerable percentage of hernias present to the doctor late leading to a higher incidence of morbidity and mortality [1]. The management of inguinal hernia poses therapeutic challenges to general surgeons practicing in resource-limited countries. Late presentation of the disease and the lack of modern therapeutic facilities such as laparoscopy and mesh are among the major drawbacks in developing countries [4].

The fact that over a hundred types of inguinal hernia repairs have been described and practiced at some point over the last century attests to the fact that none has been considered clearly superior to the others. Only three techniques remain today that have been scientifically validated and can be recommended for clinical use: (1) Shouldice’s technique (2) Lichtenstein’s open mesh hernioplasty (3) Laparoscopic posterior mesh hernioplasty. Each technique has distinct advantages and disadvantages in terms of equipment required, difficulty to master, materials, complication and recurrence, recovery time, and acute and chronic pain rates. However, in recent years, the use of mesh for inguinal hernia repair has become the norm. The reduction in recurrence rate from more than 15% with tissue repairs to less than 1% with mesh, as well as the reduction in postoperative pain and a shorter recovery time, have all contributed to the popularity and widespread use of tension-free mesh repairs. Laparoscopic inguinal hernia repair is a relatively newer modality in the surgeon's armamentarium [5].

In 1983, surgeon Ralph Ger introduced laparoscopic surgery as a method for repairing inguinal hernias [6]. Today, the most commonly used laparoscopic techniques are totally extraperitoneal (TEP) repair and transabdominal preperitoneal (TAPP) repair. Both procedures necessitate the use of general anesthesia and a synthetic mesh. TAPP necessitates peritoneal cavity access to the hernia site. The peritoneal cavity is not entered during TEP. The hernia site is instead accessed through the preperitoneal plane, and mesh is used to seal the hernia orifice outside the peritoneum. Both methods enable visualization of the entire inguinal floor, revealing any direct, indirect, or femoral hernias [7].

General surgeons who support laparoscopic repair also tout its advantages for bilateral and recurrent hernias. Bilateral hernias can be repaired simultaneously with laparoscopic repair, and undetected hernias on the opposite side can be ruled out. The TAPP technique is very effective at detecting contralateral hernias quickly [8].

Yang et al. compared laparoscopic to Lichtenstein repair of recurrent inguinal hernias in a meta-analysis of randomized controlled trials. They discovered that patients who underwent a laparoscopic procedure had lower rates of wound infection and chronic pain [9].

Köckerling et al. conducted a large prospective study. The TAPP group included 6700 subjects and the TEP group included 10,887 subjects. The surgical technique had no significant impact, according to multivariable analysis. The author concluded that there is no significant difference between TEP and TAPP in terms of intraoperative and general postoperative complication rates, as well as the reoperation rate for complications [10].

Vãrcuæ et al. conducted a comparative study of TAPP (n=36) and TEP (n=34). There were two cases of bleeding in the TAPP group; both were managed by laparoscopic sealing of the damaged vessels, eight cases of postoperative edema of the testis in TAPP and three cases in TEP. The authors concluded that differences between TEP and TAPP in the study were related to minor complications, no major complications occurred [11].

Bansal et al. in a prospective, randomized, controlled trial comparing TEP versus TAPP reported that early postoperative pain, longer operative time, and cord edema were significantly higher with TAPP, whereas the incidence of seroma formation was higher in TEP. The cost was comparable between the two [12].

Thus, various studies have reported variable outcomes with both the techniques, some of them stating them as comparable while some in favor of TEP. The choice of surgery depends in particular on various patient characteristics. In India, particularly in northern India, there is a paucity of data about the comparison of these two surgical procedures. Hence, we decided to undertake this study.

Aim and objectives

The aim of this study is to compare the clinical outcome and safety of Lap TEP and Lap TAPP for inguinal hernia repair. The objectives are: (1) to assess and compare intraoperative factors in subjects undergoing laparoscopic TEP and TAPP approaches for inguinal hernia repair, (2) to assess and compare immediate postoperative complications in subjects undergoing laparoscopic TEP and TAPP approaches for inguinal hernia repair, (3) to compare the duration of hospital stays between two groups, and (4) to compare the rate of recurrence between two groups by following up the cases for up to a period of six months.

## Materials and methods

The study was started after proper clearance by the Institutional Ethics Committee of Asian Institute of Medical Sciences (Faridabad, India). The study population was subjects with inguinal hernia diagnosed clinically. The study design is a hospital-based prospective analytical study.

The sample size is as follows:

Group 1: 45 subjects undergoing TEP laparoscopic hernia repair.

Group 2: 45 subjects undergoing TAPP laparoscopic hernia repair.

Schrenk (1996) in their study reported the mean difference in the duration of hospital stays between TAPP and TEP procedures to be 0.7 days with a pooled standard deviation of 0.11 days. The sample size calculation has been calculated using the formula proposed by Snedecor and Cochran, (1989) [13],

n = [16δ2/d2] + 1

where δ is the standard deviation and d is the mean difference between the two groups. The proposed mean difference between the two groups is 0.7 days, with a pooled standard deviation of 1.1 days. Thus, in the present study δ =0.11 and d=0.7 mm Hg. Putting these values in the formula we get the equation.

n = [{16 (1.12)}/ (0.72)] + 1

 =19.4/ 0.49 + 1

= 39.592 + 1 = 40.592~ 41 patients in each group.

Thus, the calculated sample size is 41 samples in each group. After adding 10% contingency, the sample size comes out to be 45 in each group. The duration of the study is September 2020 to September 2022.

Inclusion criteria 

Inclusion criteria were all patients diagnosed clinically as having inguinal hernias, undergoing laparoscopic mesh hernioplasty, of age between 15 and 75 years, with both unilateral and bilateral primary inguinal hernias, and who gave informed consent.

Exclusion criteria 

Exclusion criteria were patients of age less than 15 years and more than 75 years, patients not willing to follow up, patients lost or dead during follow-up, pregnant women, pre-existing port site herniation defect, patients undergoing additional procedures such as bowel resection and anastomosis, irreducible hernia, or needing emergency procedure, contraindication to lap hernia repair like known adhesions due to previous abdominal surgery, and giant hernia.

Methodology 

All patients with inguinal hernia who were treated surgically by laparoscopic mesh repair were included in the study. The preoperative, operative, and postoperative data of patients were collected on different aspects as per the defined protocol. Patients were randomly divided into two groups on the basis of surgical procedure. The first group of patients underwent laparoscopic TAPP mesh repair, and the second group of patients underwent laparoscopic TEP mesh repair. Randomization was done through a random draw of lots, i.e., a total of 90 slips with Group I and Group II inscribed on them were placed in a wide-mouthed jar and each subsequent patient scheduled to undergo hernia repair was asked to pick one slip from the jar. The patients were subsequently allocated to the corresponding study group. All procedures were performed using the standard technique for laparoscopic inguinal hernia repair. All the procedures were performed by the same surgical team.

Their intraoperative and postoperative findings like pain, time to tolerate feed, urinary retention, hospital stays, and recurrence were noted. The visual analogue scale (VAS) was used for accessing postoperative pain. Patients were followed up at regular intervals for up to six months.

Organization of work elements

Institutional ethics committee clearance was taken. Informed consent and patient information sheets were prepared. Patients were recruited in the study after due informed consent.

The parameters to be compared are the total duration of hospital stays, postoperative pain and analgesia, postoperative time of tolerating feed, and postoperative complications (minor/major) follow-up at one week, one month, and six months.

The minor complications are hematoma, seroma, wound/superficial infection, and scrotal edema, and the major complications are mesh/deep infection, port site hernia, bowel obstruction, and recurrence. Recurrences may occur at one week, one month, or six months.

## Results

Statistical analysis

Data were expressed as percentage and mean ± S.D. Kolmogorov-Smirnov analysis was performed for checking the linearity of the data. Fischer’s exact test or Chi-square test was used to analyze the significance of difference in frequency distribution of the data. Student’s unpaired t-test was used to assess the significance of difference between study groups. P value <0.05 was considered statistically significant.

Results

The majority of subjects in the TEP group were </=40 years of age, while those in the TAPP group were in the sixth decade of their life. Age distribution between the two groups was compared using the Chi-square test. No significant difference was noted between the two groups (p=0.46) (Table 1).

**Table 1:** Comparison of the mean age in two groups using Student’s unpaired t-test TEP: Totally extraperitoneal; TAPP: transabdominal preperitoneal

	Group	N	Mean	Std. Deviation	Std. Error Mean	p-value
Age (Years)	TEP	45	48.2667	16.35876	2.43862	0.485
	TAPP	45	50.6444	16.07250	2.39595	

The mean weight in the two groups was compared using Student’s unpaired t-test. Though the mean weight in TAPP subjects (67.02 ± 7.72) was found to be lower compared to the mean weight in the TEP group (67.40 ± 9.78), the difference failed to reach statistical significance (p=0.83) (Table 2).

**Table 2:** Comparison of the mean weight in two groups using Student’s unpaired t-test TEP: Totally extraperitoneal; TAPP: transabdominal preperitoneal

	Group	N	Mean	Std. Deviation	Std. Error Mean	p-value
Age (Years)	TEP	45	48.2667	16.35876	2.43862	0.485
	TAPP	45	50.6444	16.07250	2.39595	

The duration needed (in minutes) for TAPP (74.44± 17.26) was found to be significantly less (p=0.003) compared to TEP (89.22 ± 26.98) using Student’s unpaired t-test. In the TEP group, conversion to open was found to be positive for three subjects (6.7%) while it was absent in TAPP subjects (Table 3).

**Table 3:** Comparison of the duration needed for TAPP and TEP using Student’s unpaired t-test. TEP: Totally extraperitoneal; TAPP: transabdominal preperitoneal

	Group	N	Mean	Std. Deviation	Std. Error Mean	t	p-value
Duration (min)	TEP	45	89.2222	26.98812	4.02315	3.094	.003
	TAPP	45	74.4444	17.26209	2.57328		

Pain at 24 hours was found to be higher in TAPP subjects on the VAS (4.68 ± 1.74) compared to that in TEP subjects (4.24 ± 0.60), but the difference failed to reach statistical significance (p=0.110). Time to liquid diet (in hours) was found to be the same (6.26± 1.25) in both groups (Table 4).

**Table 4:** Comparison of pain at 24 hours (VAS) in TAPP subjects and TEP subjects TEP: Totally extraperitoneal; TAPP: transabdominal preperitoneal

	Group	N	Mean	Std. Deviation	Std. Error Mean	t	p-value
Pain after 24 hours (VAS)	TEP	45	4.2444	.60886	.09076	-1.615	.110
	TAPP	45	4.6889	1.74281	.25980		

Association of the duration of hospital stays with the type of surgery was performed using Fischer’s exact test. No significant difference was detected. Two subjects (4.4%) in each group needed to stay in the hospital for two days. Others were discharged the next day (Table 5).

**Table 5:** Association of the duration of hospital stays with the type of surgery TEP: Totally extraperitoneal; TAPP: transabdominal preperitoneal

		TEP	TAPP	Total
	Group 1	43 (95.6%)	43 (95.6%)	86 (95.6%)
	Group 2	2 (4.4%)	2 (4.4%)	4 (4.4)
Total		45 (100%)	45 (100%)	90 (100%)

Six subjects (13.2%) showed hematoma in the TEP group while five subjects (11%) showed hematoma in the TAPP group after one week of surgery. None showed the same at one-month and six-month follow-up visits. There was no statistical difference between the two groups (Table 6).

**Table 6:** Occurrence of hematoma in the TEP group and TAPP group after one week of surgery TEP: Totally extraperitoneal; TAPP: transabdominal preperitoneal

Characteristics	Group	1 week	1 month	6 months
Hematoma	TEP	2 (4.4%)	0 (0%)	0 (0%)
	TAPP	1 (2.2 %)	0 (0%)	0 (0%)

Eight subjects (17.6%) showed seroma in the TEP group while three subjects (15.4%) showed seroma in the TAPP group after one week of surgery. None showed the same at one-month and six-month follow-up visits (Table 7).

**Table 7:** Occurrence of seroma in the TEP group and TAPP group after one week of surgery TEP: Totally extraperitoneal; TAPP: transabdominal preperitoneal

Characteristics	Group	1 week	1 month	6 months
Seroma	TEP	4 (8.9%)	0 (0%)	0 (0%)
	TAPP	3 (6.7%)	0 (0%)	0 (0%)

Two subjects (4.4%) showed superficial wound infection in both the TEP group and TAPP group after one week of surgery. None showed the same at one-month and six-month follow-up visits (Table 8). 

**Table 8:** Wound infection in both the TEP group and TAPP group after one week of surgery TEP: Totally extraperitoneal; TAPP: transabdominal preperitoneal

Characteristics	Group	1 week	1 month	6 months
Wound infection	TEP	2 (4.4%)	0 (0%)	0 (0%)
	TAPP	2 (4.4%)	0 (0%)	0 (0%)

Four subjects each (8.9%) showed scrotal edema in the TEP group as well as the TAPP group after one week of surgery. None showed the same at one-month and six-month follow-up visits (Table 9).

**Table 9:** Occurrence of scrotal edema in the TEP group and TAPP group after one week of surgery TEP: Totally extraperitoneal; TAPP: transabdominal preperitoneal

Characteristics	Group	1 week	1 month	6 months
Scrotal edema	TEP	4 (8.9%)	0 (0%)	0 (0%)
	TAPP	4 (8.9%)	0 (0%)	0 (0%)

No subject showed port site hernia at one-week, one-month, and six-month follow-up visits without closure of the sheath. Two subjects (4.4%) each showed groin pain in the TEP group as well as the TAPP group after one week of surgery. None showed the same at one-month and six-month follow-up visits (Table 10).

**Table 10:** Incidence of groin pain in the TEP group and TAPP group after one week of surgery TEP: Totally extraperitoneal; TAPP: transabdominal preperitoneal

Characteristics	Group	1 week	1 month	6 months
Groin pain	TEP	2 (4.4%)	0 (0%)	0 (0%)
	TAPP	2 (4.4%)	0 (0%)	0 (0%)

No subject showed bowel obstruction at one-week, one-month, and six-month follow-up visits. They did not show mesh infection at one-week, one-month, and six-month follow-up visits, and also no subject showed the recurrence of hernia at one-week, one-month, and six-month follow-up visits.

## Discussion

No significant difference was noted between the two groups (p=0.46) indicating that the two groups were matched for age. The groups were also matched for weights. This indicates that both our groups were comparable with each other ruling out any selection bias or need for adjustments.

In our study, the duration needed for TAPP in minutes was found to be significantly less compared to that needed in TEP. However, in a study by Khoury et al., the average operative time was 55 min for the TAPP and 50 min for the TEP procedure. However, the difference failed to reach statistical significance [14]. Shakya et al. reported that the average operative time for unilateral TEP was 87.21±30.48 minutes (range 50-185 minutes), for bilateral TEP 120 minutes, whereas for unilateral TAPP was 95.4±12.34 min and for bilateral TAPP was 140±46.03 minutes (range 75-190 minutes) [15].

Köckerling et al. also reported that the TAPP technique had a mean operation time of 52.62±23.58 minutes. The TEP technique had a significantly shorter mean operation duration of 48.58±21.52 min [10].

All the above studies reported the operation duration to be longer in TAPP compared to TEP though some of them failed to reach statistical significance. The finding was found to be in contrast with our study. But Vãrcuæ et al. noted the duration of surgery to be longer in the TEP group (median 74 min (62-98)) compared to the TAPP group (median 69 minutes (40-135)). This study finding was in accordance with our study [11]. Similar to this study, the average duration for surgery in our study exceeded an hour. The reason for this may be that TEP requires dissection in the preperitoneal space to reach the inguinal region, whereas TAPP can approach the inguinal region directly from the abdominal route. Also, working space is limited in TEP as compared to TAPP leading to difficulty in dissection and mesh placement.

In the TEP group, conversion was found to be positive for three subjects in view of the difficulty in dissection while it was absent in TAPP subjects but the difference failed to reach statistical significance. Similarly, Schrenk et al. have reported a higher conversion rate in the TEP group in a comprehensive review [13]. Vãrcuæ et al. also noted that there was a conversion from TEP to open Lichtenstein procedure due to the lack of muscle relaxation and the inability to create a proper working space, but no conversion was reported in the case of TAPP [11]. This finding is in accordance with our study. Conversion to open may have to be done mainly when dissection is not possible laparoscopically and in such a patient both TAPP and TEP would have the same difficulty. Thus, the open conversion rate has no significant difference.

In our study, pain at 24 hours was found to be higher in TAPP subjects on VAS compared to that in TEP subjects, but the difference failed to reach statistical significance. Time to liquid diet was found to be the same in both groups, and no significant difference was thus noted. The duration of hospital stays was also noted to be similar in both groups, and two subjects from each group were found to need an extended stay of two days in the hospital; the remaining patients were discharged after one day. Similarly, Bansal et al. reported that the TAPP group required significantly more parenteral analgesia, even though the median duration of oral analgesic intake was comparable in both groups. At 6 and 24 hours after surgery, the TAPP group had significantly higher pain scores. At one-week and six-week follow-ups, the pain score in the TAPP repair group was significantly higher than in the TEP repair group [12]. However comparable pain was recorded in the study by Vãrcuæ et al. between both methods [11]. Khoury et al. reported that hospital stay was shorter for the TEP group [14]. Shakya et al. reported however that the mean hospital stay and the time to full recovery were similar in the TAPP and TEP groups [15]. TAPP and TEP essentially include the same steps except for their approach to the inguinal region. The dissection and mesh placement are in the same plane for both the procedures. Thus, postoperative pain, tolerance to diet, and length of hospital stay do not have a significant difference and a higher incidence in any one procedure may be due to chance.

In our study, it was noted that the frequency of hematoma at one week was higher in the TEP group compared to the TAPP group; also seroma was frequent in the TEP group (17.6%) compared to the TAPP group (15%) at one week. The rate of wound infection and scrotal edema was similar in both groups at one week. At one month and six months, there was no difference between two groups with respect to all these complications. None of the subjects reported port site hernia, bowel obstruction, mesh infection, or recurrence during the six-month follow-up. Similarly, Khoury et al. reported that there were no intraoperative complications. The postoperative complications were comparable in both groups. Also, the recurrence rate and the return to normal activity were not different between the two types of repair [14]. Köckerling et al. in the study with a large sample size reported that there are significant differences between the two surgical techniques in terms of intraoperative complications [10]. Secondary bleeding occurred more frequently after the TEP operation, while seroma was seen more commonly after the TAPP operation. However, the difference in the postoperative complication rate between TEP and TAPP did not result in any difference in the reoperation rate due to surgical complications. Bansal et al. reported that though no major complications occurred in any of the groups, the TAPP group had a higher incidence of cord edema, which was statistically significant [12]. None of the patients had a residual seroma at the three-month follow-up. There was no statistically significant difference in the incidence of wound infections between the two groups. The difference in the incidence of postoperative seroma and hematoma may be because TEP requires more extensive dissection to reach its target as compared to TAPP which directly approaches the posterior wall of the inguinal canal intraabdominally. The raw surfaces created during TEP dissection may increase the chances of seroma and hematoma. Thus, results of all these studies were in accordance with our study.

## Conclusions

TAPP and TEP have comparable postoperative outcomes. There is no clear superiority of one procedure over the other with respect to postoperative pain, length of hospital stay, hematoma, seroma, surgical site infection, scrotal edema, port site hernia, bowel obstruction, mesh infection, and recurrence of hernia. However, operative time was less for TAPP as compared to TEP.
